# Cognitive rescue in aging through prior training in rats

**DOI:** 10.18632/aging.204808

**Published:** 2023-06-19

**Authors:** Alexandra Gros, Szu-Han Wang

**Affiliations:** 1 Centre for Clinical Brain Sciences, The University of Edinburgh, Chancellor’s Building, Edinburgh, Scotland, UK

**Keywords:** memory consolidation, reconsolidation, memory modulation, lifelong training, cognitive stimulation

## Abstract

Cognitive decline in spatial memory is seen in aging. Understanding affected processes in aging is vital for developing methods to improve wellbeing. Daily memory persistence can be influenced by events around the time of learning or by prior experiences in early life. Fading memories in young can last longer if a novel event is introduced around encoding, a process called behavioral tagging. Based on this principle, we asked what processes are affected in aging and if prior training can rescue them. Two groups of aged rats received training in an appetitive delayed matching-to-place task. One of the groups additionally received prior training of the same task in young and in mid-life, constituting a longitudinal study. The results showed long-term memory decline in late aging without prior training. This would reflect affected encoding and consolidation. On the other hand, short-term memory was preserved and novelty at memory reactivation and reconsolidation enabled memory maintenance in aging. Prior training improved cognition through facilitating task performance, strengthening short-term memory and intermediate memory, and enabling encoding-boosted long-term memory. Implication of these findings in understanding brain mechanisms in cognitive aging and in beneficial effects of prior training is discussed.

## INTRODUCTION

Aging is a natural biological process and is often associated with a decline in cognitive function [1]. Age-related impairment in spatial [2, 3] and episodic memory [4–6] is common in humans. Similarly, deficits in navigational strategy, spatial memory, pattern separation and reduction in working memory capacity are also observed during normal aging in animals [7–14]. Such impairment tends to occur at a later stage of aging (e.g., > 24 months in rodents). We recently showed that an appetitive delayed matching-to-place task in rats, simulating an everyday spatial memory in humans, can sensitively reveal cognitive impairment at an earlier stage (12–13 months old) [15]. This model can allow earlier detection of cognitive aging and provide much needed insight into understanding the memory processes and underlying mechanisms that are first affected in aging.

Memory persistence relies on a highly dynamic cognitive process involving memory encoding, modulation, consolidation and reconsolidation [16]. Encoding of a weak event does not lead to consolidation of a long-lasting memory. However, unrelated novelty, such as exploration in a new environment and/or in a box with new substrates, introduced around encoding of a weak event can enable the persistence of long-term memories [17, 18]. This resembles how we remember details of personal circumstances when encountering significant events or hearing shocking news, a phenomenon called flashbulb memories [19, 20]. This process in which novelty enhances memory persistence follows similar principles that are first reported in the synaptic tagging and capture hypothesis [21], and is called behavioral tagging [22, 23] The synaptic tagging and capture theory proposes that plasticity-related proteins are involved in generating long-lasting changes when they are captured by synaptic tags after synaptic stimulation [24]. Behavioral tagging represents a robust method to enable longer persistence of everyday memories [18, 25] that normally fade away over time when no peri-encoding novelty or other memory modulating events are introduced. While novelty around the time of encoding supports persistence of various types of long-term memory in young [22], it is not as effective in early cognitive aging. Novelty around encoding improves the persistence of intermediate-term (6 h) memory, but not long-term (24 h) memory in middle-aged rats [15, 26]. This study aimed to further investigate which components of the dynamic cognitive processes from task acquisition, encoding, consolidation, to behavioral tagging are further deteriorated in advanced aging. Memory tasks that involve updating daily spatial information provide an advantage for longitudinal studies in that these tests can be repeated over the course of aging.

An important factor to consider in aging is cognitive stimulation in promoting memory performance. The lifestyle-cognition hypothesis holds that maintaining an active lifestyle and engaging in intellectual, physical, and social activities during one’s life help prevent cognitive decline during aging [27, 28]. Behavioral interventions that aim to protect brain functions against age-related decline are often described in the form of cognitive training as a non-pharmacological treatment for cognitive decline during normal aging [29].

Here we asked if cognitive training in young and mid-life would improve cognitive aging and which elements of the cognitive processes at old age are preferentially protected through such training. Specifically, we examined the errors and speed in task acquisition, and the presence or absence of short-term memory, intermediate-term memory, long-term memory, post-reactivation long-term memory, and novelty-enhanced memory persistence in advanced aging.

To this end, we trained two groups of rats in an appetitive delayed-matching-to-place task in the event arena. The first group was trained, and memory assessed at an older age (19–23 months old) only (i.e., no prior training at younger ages). The second group received the same training at an older age (19–23 months old) but also underwent prior training when they were young (3–5 months old), and when they were middle-aged (11–13 months old). We found that prior training, simulating active intellectual and physical activities in humans, improves several aspects of learning and memory and the findings provide insights on underlying processes and mechanisms for improving cognitive aging.

## RESULTS

### Training performance in old rats without or with prior training

**Figure 1:**
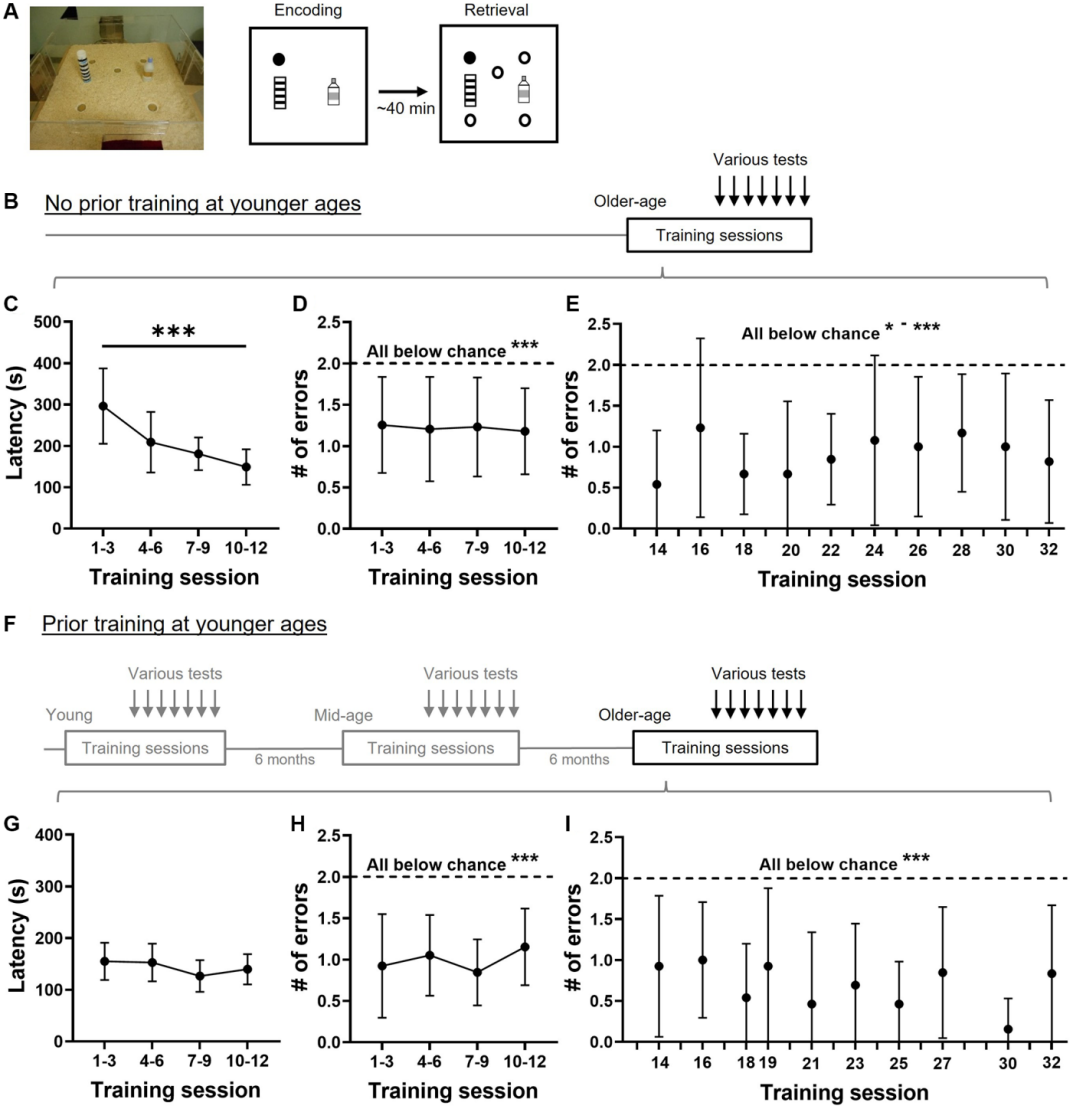
**Training performance in the appetitive spatial task.** (**A**) Event arena and training paradigm. The appetitive spatial task was composed of two trials per session. During the encoding trial, the rat found hidden food rewards (filled circle) inside the arena. After a delay, rats would encounter 5 different sandwells (circles) with the same location being rewarded. (**B**) A group of aged rats (19–23 months old) were trained at the older age followed by interleaving training and probe sessions. Various tests refer to the memory tests presented in Figures 2–5. (**C**) Latencies to retrieving rewards gradually decline across 4 blocks of training (one-way, repeated measure ANOVA, F_3, 12_ = 18.8, *p* < 0.0001). (**D**) The number of errors made at retrieval was below chance (dashed line; one-sample *t*-tests, all *p* < 0.001) and stable across 4 blocks of training (one-way, repeated measure ANOVA, F_3, 12_ = 0.06, *p* = 0.96). (**E**) The number of errors made at retrieval during interleaving training was below chance throughout the study (all *p* < 0.05–0.001). (**F**) A second group of rats was trained and tested in young (3–5 months old), in middle age (11–13 months old), and at later age (19–23 months old). Various tests at the older age refer to the memory tests presented in Figure 2–5. (**G**) Latencies to retrieving rewards were stable (one-way, repeated measures ANOVA, F_3, 12_ = 2.31, *p* = 0.11). (**H**) The number of errors made at retrieval was below chance (dashed line; all *p* < 0.0001) and stable across 4 blocks of training (one-way, repeated measures ANOVA, F_3, 12_ = 0.89, *p* = 0.44). (**I**) The number of errors made at interleaving retrieval trials was below chance (all *p* < 0.002). All data are presented as mean ± SD. ^*^p < 0.05, ^**^p < 0.01, ^***^p < 0.005.

The rats were trained in an appetitive delayed-matching-to-place (ADMP) task to encode the reward location and to navigate to find more rewards at the matching location after a delay in an open arena (Figure 1A). The regular daily training session consisted of an encoding trial followed by a retrieval trial (with 4 unrewarded locations and 1 rewarded location that matched to the encoding one). The encoding trial constituted the opportunity to register where food was available in the arena in that session. During the retrieval trial, the rat could use place memory to navigate effectively to find more rewards after a delay.

In old rats without prior training, the latency to retrieve all rewards decreased over the 4 blocks of training sessions (Figure 1C). The statistical power of latency reduction from block 1 to block 4 was 1 and the Cohen’s d was 1.6. The number of errors was stable over the 4 blocks of training (Figure 1D) and significantly below chance. To our surprise, the number of errors was below chance from the first block of training (one-sample *t*-test, t_12_ = 4.62, *p* = 0.0006), and even from the first session of training (one-sample *t*-test, t_12_ = 2.9, *p* = 0.013). This may suggest that older rats would use a ‘matching’ rule to learn the task from the beginning. Interleaving training sessions were introduced between probe tests and the errors made in these retrieval trials remained significantly below chance (Figure 1E), showing stable performance of the task.

In old rats with prior training at young and middle age, the latency to retrieve all rewards remained stable over the 4 blocks of training (Figure 1G). The number of errors also remained stable across 4 blocks of initial training (Figure 1H) and were significantly below chance. The errors they made during interleaving training sessions was also significantly below chance (Figure 1I).

Comparisons between these two groups indicated shorter latency in the group with prior training at blocks 1 and 3. A two-way ANOVA on Figure 1C and 1G revealed a significant prior training effect (F_1, 24_ = 21.4, *p* = 0.0001; Bonferroni’s multiple comparisons test, block 1, *p* = 0.0004; block 2, *p* = 0.096; block 3, *p* = 0.003; block 4, *p* > 0.99). The interaction between the prior training factor and the training block was also significant (F_3, 72_ = 10.58, *p* < 0.0001). These suggest that prior training contributes to shorter latencies compared to the group without prior training and the effect is stronger at the early training phase. A two-way ANOVA on Figure 1D and 1H showed only a trend of prior training effect (F_1, 24_ = 3.4, *p* = 0.078), suggesting that prior training has a mild effect on reducing the errors during early training. A two-way ANOVA on Figure 1E and 1I showed an insignificant prior training effect (F_1, 24_ = 1.15, *p* = 0.3), suggesting that prior training does not reduce errors further after the animals have gone through numerous training sessions.

The group with prior training represented a longitudinal study that allows comparison across lifespan from young and middle-age to an older age. Data from young and middle-aged animals were published in our previous article [15]. To understand if there is saving from young to older ages, we performed several analyses on latencies and errors. On latencies in Figure 1G here and in Figure 1D and 1F in our previous study [15], a two-way ANOVA showed a significant age effect (F_1.59, 19.05_ = 79.25, *p* < 0.0001). The within-group saving on latencies in the first training block was most significant from young to mid-age (i.e., young, mean ± SD, 285.3 ± 63.4 s vs. mid-age, 118.9 ± 34.5 s, Tukey test, *p* < 0.0001), with no further saving from mid-age to old (mid-age vs. old age, mean ± SD, 155.05 ± 35.94 s, Tukey test, *p* = 0.28). When comparing errors in Figure 1H and in Figure 1C and 1E in our previous study [15], the age effect was also significant (two-way ANOVA, F_1.96, 24_ = 8.27, *p* = 0.002). The within-group saving on errors in the first training block was also most apparent from young to mid-age (young, mean ± SD, 1.5 ± 0.8 vs. mid-age, 0.7 ± 0.5, Tukey test, *p* = 0.03), and not from mid-age to old (mid-age vs. old age, mean ± SD, 0.9 ± 0.6, Tukey test, *p* = 0.99). These would suggest that once the animals acquire the task in early life, they can relearn and perform the task effectively and fairly accurately in an older age.

### Short-term memory is intact in old rats, is facilitated by prior training, and remains allocentric

**Figure 2:**
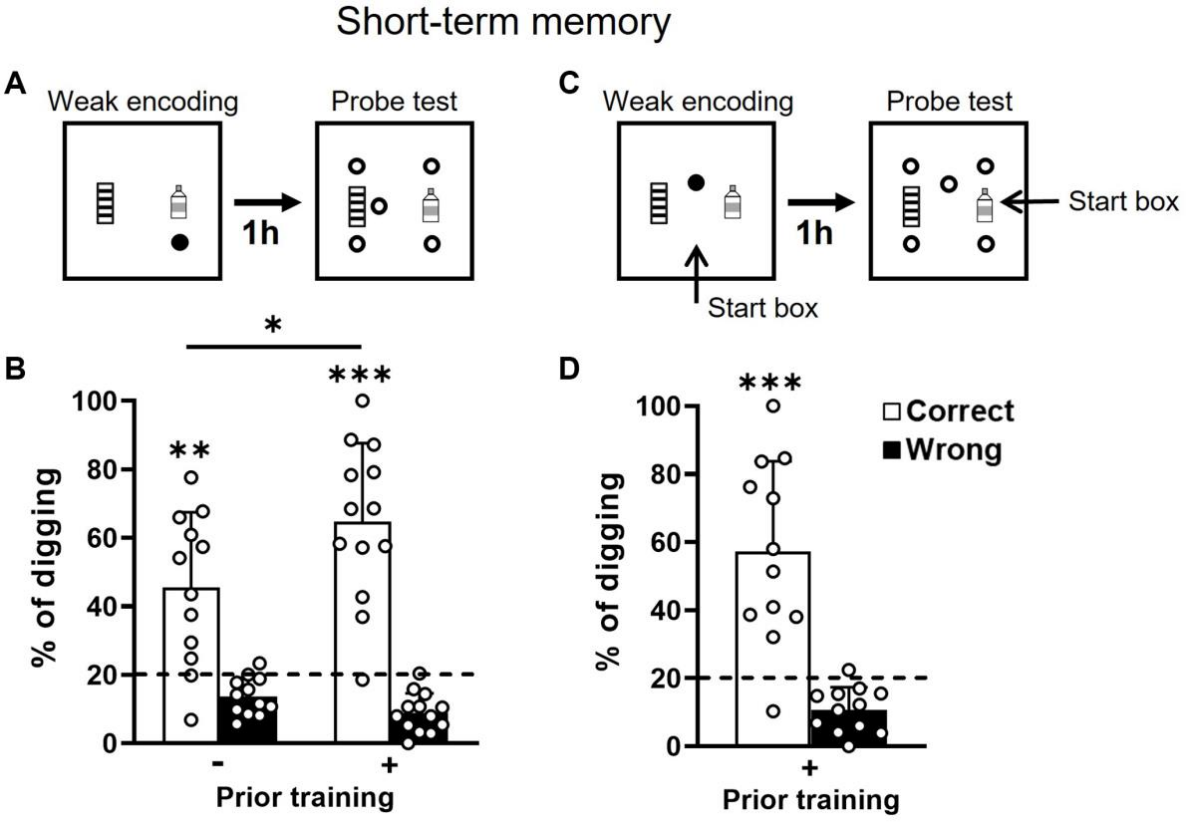
**Short-term retention of everyday spatial memory in aging.** (**A**) Rats received a weak encoding (1 reward, filled circle). One hour later, they were tested in a probe trial with 5 non-rewarded sandwells (open circles). (**B**) The percentage of correct digging was significantly above chance (dashed line; one-sample *t*-test, no prior training, t_11_ = 4.01, *p* = 0.002, attrition of 1 rat; priorly trained, t_12_ = 7.04, *p* < 0.001) after weak encoding in both groups and the group difference was significant (unpaired *t*-test, t_23_ = 2.14, *p* = 0.043). The statistical power of short-term memory in the group without prior training was 0.99 and the Cohen’s d was 1.16. (**C**) Similar to procedures in A except that the start location was changed at the probe test. (**D**) The percentage of correct digging was significantly above chance (dashed line; one-sample *t*-test, t_11_ = 4.86, *p* < 0.001). Data are presented as mean ± SD. ^*^p < 0.05, ^**^p < 0.01, ^***^p < 0.005.

To assess the short-term memory, rats would receive a weak (1 reward) encoding trial followed by a probe test in which five nonrewarded sandwell would be presented at a 1 h delay (Figure 2A). The percentage of time spent in digging the correct location over total digging time of all locations constituted the % of correct digging which was used as the index of memory.

The group with prior training showed significantly better performance than the group without prior training (Figure 2B). This would indicate that prior training facilitates short-term memory in old rats. No group difference was observed with other measurements (unpaired *t*-tests: errors, *p* = 0.29; latency to encoded location, *p* = 0.23; latency to collect all rewards after the probe, *p* = 0.41). The short-term memory was significantly above chance in both groups (Figure 2B), indicating a good short-term memory. Associated with our previous results showing that short-term memory was not impaired in young and middle-aged rats, these results indicated that short-term memory is not affected by aging in this ADMP task.

To determine if an allocentric strategy was used to find the rewards as seen in young rats [18], the start box was changed between the encoding trial and the probe test in the group with prior training (Figure 2C). The correct digging percentage remained significantly above chance after changing the start box (Figure 2D) and was indifferent from the percentage when the start box was unchanged (Figure 2B right, paired *t*-test, t_11_ = 0.84, *p* = 0.42). This suggests that old rats can use an allocentric strategy and spatial cues to find the correct sandwell.

### Intermediate-term memory is facilitated by prior training in old rats

**Figure 3:**
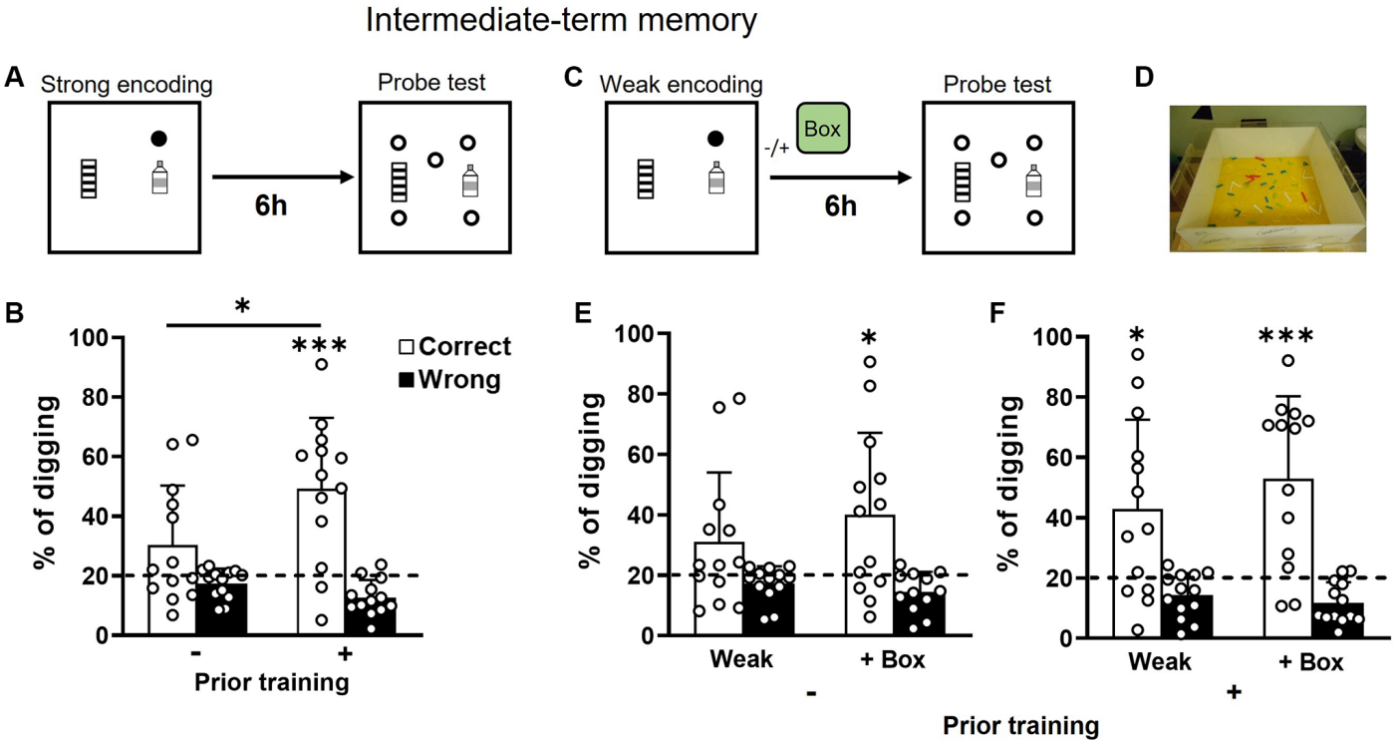
**Intermediate-term retention of everyday spatial memory in aging.** (**A**) Rats received a strong encoding trial (3 rewards, filled circle). Six hours later, they were tested in a probe trial with 5 non-rewarded sandwells (open circles). (**B**) The percentage of correct digging was not significantly above chance (dashed line) in older rats without prior training (one-sample *t*-test, t_12_ = 1.88, *p* = 0.085) but significantly above chance in older rats with prior training (one-sample *t*-test, t_12_ = 4.45, *p* < 0.001). The group difference was significant (unpaired *t*-test, t_24_ = 2.2, *p* = 0.04). (**C**) Similar to procedures in A except that exploration in a novel box (green box) was introduced or omitted at 30 min after a weak encoding trial (1 reward). (**D**) An example of a novel box. (**E**) In rats with no prior training, the percentage of correct digging was not significantly above chance (dashed line; one-sample *t*-test, t_12_ = 1.74, *p* = 0.11) after weak encoding and was significantly above chance after weak encoding with novelty (one-sample *t*-test, t_11_ = 2.89, *p* = 0.015). No difference was observed between the absence or presence of novelty (paired *t*-test, t_11_ = 0.87, *p* = 0.4). (**F**) In rats with prior training, the percentage of correct digging was significantly above chance (dashed line) in both conditions (absence of novelty: one-sample *t*-test, t_12_ = 2.8, *p* = 0.02; presence of novelty: one-sample *t*-test, t_12_ = 4.34, *p* = 0.00). No difference was observed between the absence or presence of novelty (paired *t*-test, t_12_ = 0.99, *p* = 0.34). Data are presented as mean ± SD. ^*^p < 0.05, ^***^p < 0.005.

With strong encoding (3 rewards, Figure 3A), intermediate-term memory (6 h) was significantly better in the group with prior training (Figure 3B). Performance was not significantly above chance in the group without prior training (Figure 3B left), but it was in the group with prior training (Figure 3B right). With weak encoding (1 reward, Figure 3C), intermediate-term memory was not significantly above chance in the group without prior training (Figure 3E left). Novelty (Figure 3D) introduced after weak encoding enabled the memory retention (Figure 3E right). The difference between the two conditions was insignificant (Figure 3E left vs. right). In the group with prior training, the percentage of correct digging was significantly above chance in both conditions (Figure 3F). The difference between the two conditions was insignificant (Figure 3F left vs. right). A two-way ANOVA on percentage of correct digging in Figure 3E and 3F showed an insignificant novelty effect (F_1, 24_ = 2.23, *p* = 0.15, partial η2 = 0.03), prior training effect (F_1, 24_ = 2.18, *p* = 0.15, partial η2 = 0.05 which was close to a medium effect), or interaction (F_1, 24_ = 0.006, *p* = 0.94, partial η2 < 0.0001). These results suggest that prior training facilitates the retention of intermediate-term memory after strong encoding in old rats.

### Long-term memory after one encoding trial is impaired at an old age, while prior training with additional encoding can rescue the impairment

**Figure 4:**
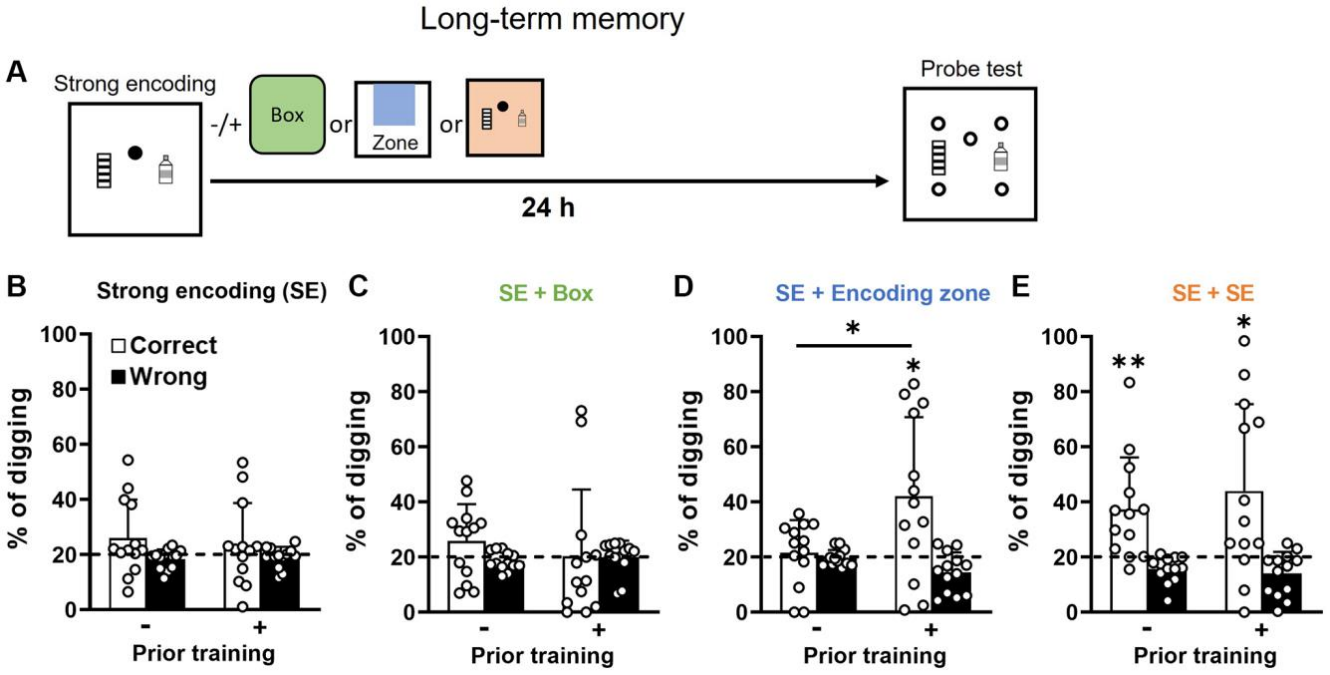
**Long-term retention of everyday spatial memory in aging.** (**A**) Rats received a strong encoding (3 rewards, filled circle) trial and 30 min later different memory-modulating events. The novel box was represented in green, the encoding zone was represented in blue, and the second strong encoding trial was represented in orange. Twenty-four hours after encoding, they were tested in a probe trial with 5 non-rewarded sandwells (open circles). (**B**) After strong encoding, the percentage of correct digging was not different from chance (dashed line) in both groups (No prior training: one-sample *t*-test, t_12_ = 1.58, *p* = 0.14; Priorly trained: t_12_ = 0.87, *p* = 0.40). No difference was observed between the two groups (unpaired *t*-test, t_24_ = 0.43, *p* = 0.67) (**C**) Novel box exposure after encoding did not lead to above-chance performance in both groups (No prior training: one-sample *t*-test, t_12_ = 1.59, *p* = 0.14; Priorly trained, t_12_ = 0.05, *p* = 0.96). No difference was observed between the two groups (unpaired *t*-test, t_24_ = 0.73, *p* = 0.48). (**D**) Exploration in the encoded zone after encoding increased the percentage of correct digging in priorly trained group only (No prior training: one-sample *t*-test vs. chance, t_12_ = 0.47, *p* = 0.65; Priorly trained: one-sample *t*-test vs. chance, t_12_ = 2.76, *p* = 0.017). A significant difference was observed between groups (unpaired *t*-test, t_24_ = 2.37, *p* = 0.03). (**E**) With a second strong encoding trial, the percentage of correct digging was significantly above chance in both groups (No prior training: one-sample *t*-test, t_12_ = 3.31, *p* = 0.006; Priorly trained: t_12_ = 2.74, *p* = 0.02). No significant group difference was observed (unpaired *t*-test, t_24_ = 0.65, *p* = 0.52) Data are presented as mean ± SD. ^*^p < 0.05, ^**^p < 0.01.

With strong encoding (3 rewards), it has been previously shown that long-term memory (24 h) was intact in young animals [15, 18] but degraded at mid-age [15]. Here we found that long-term memory after strong encoding in old rats with or without previous training (Figure 4A) was absent (Figure 4B). No difference was observed between the two groups. With this result, we confirmed that aging impairs long-term memory retention. As in middle-aged rats [15], novelty, introduced after strong encoding (Figure 4C), did not improve long-term memory in both groups (Figure 4C vs. 4B, paired *t*-test, no prior training, t_12_ = 0.04, *p* = 0.97; priorly trained, t_12_ = 0.52, *p* = 0.61), and the correct digging percentage was not higher than chance. The long-term memory after strong encoding with novelty in old rats was comparable with middle-aged rats (Figure 4C in our previous study [15], paired-*t*-test, t_13_ = 0.2, *p* = 0.84; unpaired-*t*-test, t_27_ = 1.1, *p* = 0.29).

Our previously observation showed that early aging primarily affects encoding as re-exposure to the encoding zone enables memory persistence [15]. Here, we asked if re-exposure to the encoding zone without rewards would enable memory persistence in later aging. The result showed that this was no longer effective for the group without prior training (Figure 4D left; Figure 4D left vs. 4B left, paired *t*-test, t_12_ = 0.85, *p* = 0.41). However, old rats with prior training showed higher correct percentage of digging when re-exposed to the encoding zone (Figure 4D right).

Finally, when both groups received another strong encoding trial after the initial encoding, both showed higher correct digging percentages than chance (Figure 4E), or than one encoding only condition (Figure 4E vs. 4B, paired *t*-test, no prior training, t_12_ = 2.87, *p* = 0.01; priorly trained, t_12_ = 2.18, *p* = 0.05). No significant group difference was observed after two strong encoding (Figure 4E left vs. right). Taken together across 4 panels in Figure 4, these results suggest that both encoding and consolidation after one encoding trial are affected at later aging (compared to our previous results obtained in young and middle-aged rats [15, 18, 30]). Prior training rescues long-term memory retention and renders memory processing in old rats alike which in middle-aged rats [15].

### Novelty improves memory persistence through memory reactivation and reconsolidation in old rats

**Figure 5:**
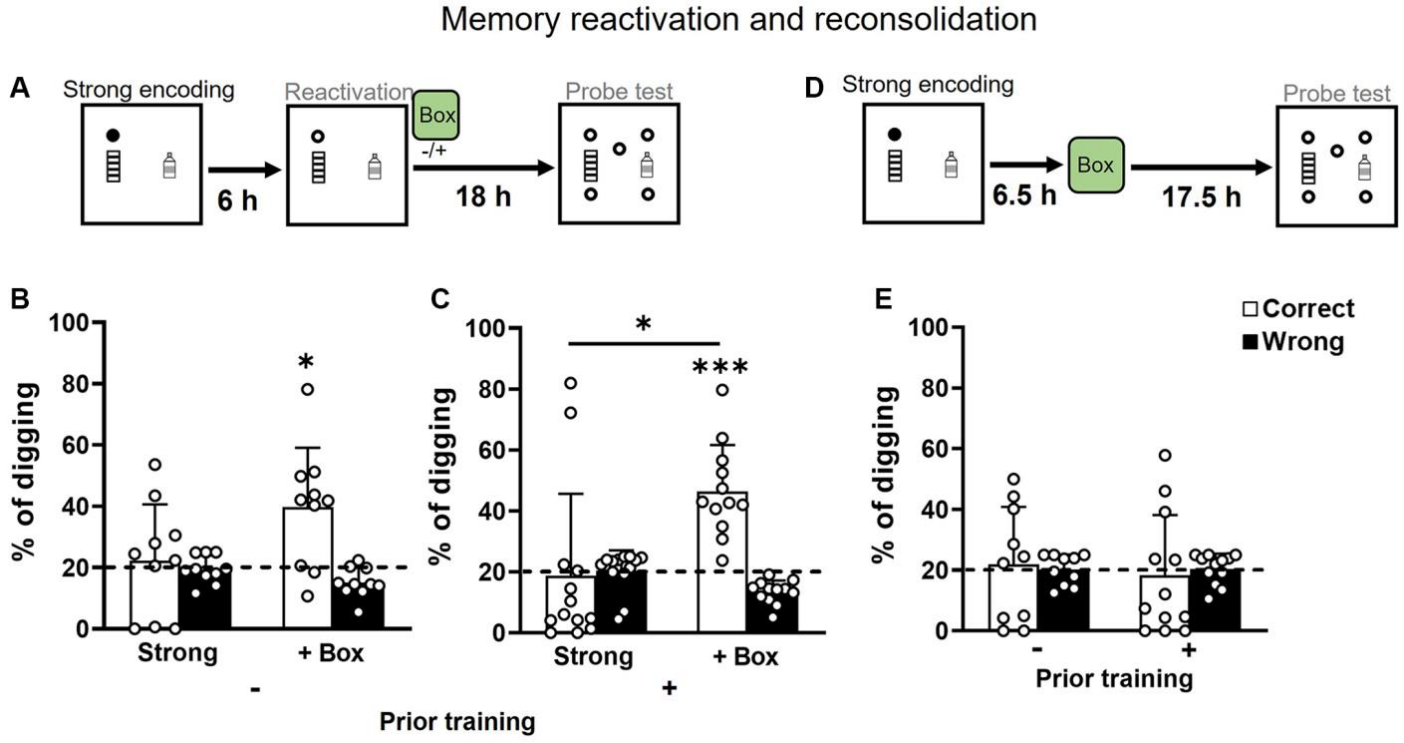
**Memory retention after strong encoding and non-rewarded reactivation in aging.** (**A**) Rats received a strong encoding trial (3 rewards, filled circle), a reactivation trial with a non-rewarded sandwell (open circle) at 6 hours later, and a non-rewarded probe trial at another 18 hours later. Exploration in a novel box was introduced or omitted at 30 min after reactivation. (**B**) In rats without prior training, the percentage of correct digging was not different from chance (dashed line) without novelty (one-sample *t*-test, t_9_ = 0.41, *p* = 0.69, attrition of 2 rats) and was significantly above chance with novelty (one-sample *t*-test, t_9_ = 3.21, *p* = 0.01). No difference was observed between the conditions (paired *t*-test, t_9_ = 1.91, *p* = 0.09; Bonferroni’s multiple comparison test *p* = 0.18). (**C**) In rats with prior training, the percentage of correct digging was not different from chance (dashed line) without novelty (one-sample *t*-test, t_12_ = 0.18, *p* = 0.86) and was significantly above chance with novelty (one-sample *t*-test, t_11_ = 6.01, *p* < 0.001). The condition difference was significant (paired *t*-test, t_11_ = 2.84, *p* = 0.02; Bonferroni’s multiple comparison test *p* = 0.014). (**D**) Rats received a strong encoding trial (filled circle), exploration in a novel box at 6.5 hours later, and a non-rewarded probe trial at another 17.5 hours later. (**E**) The percentage of correct digging was not above chance in either group (No prior training: one-sample *t*-test, t_9_ = 0.09, *p* = 0.93; Priorly trained: one-sample *t*-test, t_11_ = 0.31, *p* = 0.76). Data are presented as mean ± SD. ^*^p < 0.05, ^***^p < 0.005.

Both groups received strong encoding and, 6 h later, a non-rewarded trial to reactivate the memory that was (or was not) followed by exploration in a novel box. They were then tested in a probe trial 18 h after reactivation (Figure 5A). With reactivation only, memory in both groups was not significantly above chance (Figure 5B and 5C left). With novelty after reactivation, memory in both groups was significantly above chance (Figure 5B and 5C right). A two-way ANOVA on percentages of correct digging in Figure 5B and 5C showed a significant novelty effect (F_1, 20_ = 11.09, *p* = 0.003), an insignificant prior training effect (F_1, 20_ = 0.12, *p* = 0.73), and an insignificant interaction (F_1, 20_ = 0.51, *p* = 0.48). Novelty without reactivation (Figure 5D) did not lead to significantly higher correct digging percentages than chance in both groups (Figure 5E). These results indicate that novelty after reactivation facilitates memory persistence as seen in young [30] and in middle-aged rats [15].

## DISCUSSION

Using the appetitive delayed matching-to-place paradigm, similar to human experiences of daily encoding and retrieval of spatial information, long-term memory decline is revealed in early [15] and advanced aging (Figure 4B). Critically, prior training rescues cognition at an older age with improved learning efficiency, stronger short-term memory, stronger intermediate-term memory, and facilitated long-term memory via re-encoding (Figures 1–4). Re-exposure to the encoding zone without rewards is sufficient to rescue long-term memory in early aging [15] but not in advanced aging without prior training (Figure 4D), suggesting decline in encoding and consolidation at an older age. Novelty after reactivation at an intermediate delay enables memory persistence (Figure 5), supporting facilitation of memory through reconsolidation. Of note, this line of research in investigating the life-course changes in learning and memory of this spatial task primarily uses male animals. This is to be consistent with the decade-long efforts in using an appetitive model in understanding how behavioral tagging modulates memory persistence [15, 18, 25, 26, 30, 31]. Future research would be required to investigate if similar age-dependent changes are observed in female animals or in other behavioral paradigms.

Young rats show good long-term memory performance after strong, but not weak, encoding in this ADMP task [15, 18, 26, 30]. Novelty, introduced after encoding, can enhance the persistence of long-term memory, a phenomenon called behavioral tagging and capture. The comparison between young and middle-aged rats already showed an age-related decline in memory persistence [26]. After strong encoding, both middle-aged rats [15, 26] and older rats (Figure 4B) do not show long-term memory. Re-exposure to the encoding zone after strong encoding is sufficient to rescue to memory in middle-aged rats [15], but not in older rats (Figure 4D left), suggesting an encoding impairment from early aging that sustained to later aging. Only when both encoding and consolidation processes are reinforced with a second strong encoding trial that long-term memory is observed in old rats (Figure 4E left). It has been shown that aged animals can be divided into aged impaired vs. aged unimpaired populations based on their memory performance [32–35]. Our data in Figure 4E may suggest that certain rats outperform others. While this holds true for this memory test, the same rats do not always show superior memory in other tests. For example, among the 5 top performers in the prior training group in the two strong encoding condition (Figure 4E right), 2 of them performed consistently above group average in another similar test condition (Figure 4D right). Among the 4 top performers in the group without prior training (Figure 4E left), 2 of them performed consistently above group average in another similar test condition (Figure 4D left). This would suggest that while there is a clear individual difference in the aging population, within-subject variation also exists and requires systematic investigations in the future.

### Mechanisms underlying memory decline in aging

Through intervention at different time point of learning a task, it is established that encoding and consolidation are required for long-term memory formation [16, 36]. In early adulthood, these processes work seamlessly to achieve memory persistence. Any step that goes wrong in these processes can lead to observed memory decline or impairment. Earlier research showed long-term memory decline in old animals (e.g., 19–24 months old rodents) in spatial reference memory in the water maze [37–41], Barnes maze [42], radial maze [42–44] and in delayed-matching-to-place memory in the water maze [45, 46]. On the other hand, short-term memory is shown preserved in the radial maze in 26-month-old rats [43, 47]. Our results are consistent with this pattern of observation. These might be interpreted as intact encoding and impaired consolidation in the aging. However, if this were the case, then strengthening encoding would not rescue long-term memory while strengthening consolidation would. To test the encoding view, re-exposer to the familiar encoding zone is shown to improve memory in early [15], but not in late aging (Figure 4D left). Re-exposure to the encoded zone is designed to re-engage the encoding process without rewards. This would likely re-engage the tagging mechanisms more than the production of plasticity-related proteins as novelty or rewards are not introduced. Older rats with prior training act like middle-aged rats under this condition [15], suggesting rejuvenation of the memory function. To test the consolidation view, peri-learning novelty, postulated to generate plasticity-related proteins [17, 24, 48] to strengthen consolidation, is shown not to rescue long-term memory decline in both early [15, 26] and late aging (Figure 4C). Together, these indicate an encoding impairment in early aging and impairment in both encoding and consolidation in late aging. The implication in impairment in synaptic tagging and capture preceding changes in production of plasticity-related proteins in aging has been discussed [15].

While our approaches are based at the behavioral level of observation, aging-related changes in cognitive processes have significant implication in identifying the underlying molecular mechanisms. It may seem unusual that encoding for long-term memory is impaired while short-term memory is intact in aging when the ‘encoding’ process is believed to be a fundamental process that precedes short-term and long-term memory [49]. At the molecular level, encoding can trigger a cascade of signal induction and transduction [50]. For initial induction, findings have consistently suggested the importance of glutamatergic receptors, such as NMDA and AMPA receptors in forming short- and long-term memory [51, 52]. However, partially dissociative molecular mechanisms for short-term and long-term memory have been reported. In systematic research using post-training drug infusion in the hippocampus, inhibition of mitogen activated protein kinase (MAPK) or agonism of serotonin, 5-HT-1A, receptors impairs short-term memory selectively, but not long-term memory. On the other hand, inhibition of Ca2+/calmodulin-dependent protein kinase II (CaMKII), protein kinase C, or protein kinase G impairs long-term memory, but not short-term memory [53]. Our findings on cognitive aging, combining with molecular evidence, can provide insights on promising targets to improve memory function. Short-term memory remains intact in several rodent studies [43, 47], which corresponds with some human observation [54–57]. Boosting mechanisms that are associated to short-term memory, such as age-dependent change in 5-HT-1A receptor densities, binding sites, or its G-protein-activating capacity [58], would likely be less effective in improving long-term memory decline in aging, albeit their involvement in memory processes in young [59, 60]. On the other hand, synaptic tagging and capture research has suggested CaMK involvement in tag setting [61, 62] and it could be that targeting aging-dependent changes in this pathway [63, 64] can improve long-term memory in early aging. Aging affects synthesis and regulation of plasticity-related genes and proteins [65–67]. Postsynaptic density protein 95, is reduced in aged hippocampus synaptosomes and the protein level is correlated with spatial performance [68]. Phosphorylation of cAMP-response element binding proteins in the hippocampus is also reduced with aging [69]. When aging is more advanced, targeting molecular pathways involved in both tagging and production of plasticity-related proteins [70, 71] will be promising in improving the memory function.

At the cellular level, memory encoding and novel events increase the number of hippocampal cells, mainly neurons, that express immediate early genes in young animals [26, 72]. Effective events that lead to behavioral tagging and capture also engage a clear overlapping hippocampal population in CA1 and CA3 [72], particularly in distal CA1 and proximal CA3 when using the same behavioral task as seen in this study [26]. In early aging, encoding- and novelty-triggered neuronal populations and an overlapping population of the two in CA1 are significantly reduced [26]. A more profound reduction in the encoding population is strongly associated with the reduced overlapping population [26]. Based on behavioral and cellular evidence, encoding and associated processes are preferentially affected in early aging [15, 26].

### Prior training benefits in cognitive aging and related mechanisms

Cognitive reserve theory describes how early life activities prevent age-associated cognitive decline. This view holds that higher levels of education, engagement in leisure activities, and innate intelligence provides cognitive reserve that contributes to less cognitive decline in aging [73] or in dementia [74]. Here we show that prior training in young and in midlife, with long intervals (2 × 6 months) of no training, is sufficient to improve cognitive function in later life. It enables encoding-facilitated long-term memory (Figure 4D) and improves intermediate-term memory in old animals (Figure 3B), at a level similar to or better than middle-aged animals that are priorly trained [15]. Previous studies have shown that repeated learning experience could prevent age-related impairment in spatial reference memory [75–78]. However, there is evidence that prior training can work in a task-specific manner as aged rats that are trained in a water maze or a radial maze still show impairment in acquiring passive avoidance or a cross-maze task [76, 79].

In task acquisition, prior training provides saving in retrieval efficiency in subsequent learning at an older age (Figure 1G). In humans, spatial training improves subsequent navigation performance in young and in old participants and potentially points to a trend in preventing mild reduction of hippocampal volume over time [80]. Previous studies show that procedural memories acquired at a young age are resistant to age-related decline. In a water maze task, priorly trained aged animals show shorter latencies to relearn the task, compared to when they are first trained at a younger age [75–78]. The shorter latency in the group with prior training (Figure 1G vs. 1C) in our task is consistent with these reports on the view that procedural aspects of learning being retained in the aging process. The spatial aspect of the learning, when inferred from the ‘error’ index as a proxy, is only mildly restored by prior training (Figure 1H vs. 1D).

Better short-term memory is also seen in old animals with prior training (Figure 2B). As the number of errors and latencies are comparable between groups at the short-term memory test, one possible explaining for prior training in enabling better short-term memory is through increase the ‘certainty’ of the encoded location. It is likely that old rats with prior training would be more persistent at digging at the encoded location which then leads to increased correct performance. This could also be related to strengthened knowledge of the matching rule that they use from the onset of the task. Although we could not directly gauge how ‘certain’ the animal registers the location or the matching rule, one way to test this speculation in the future is to see if differential rates of extinction (of the encoded information or the matching rule) would be observed between old animals with or without prior training.

In young animals, once the initial training phase is concluded, subsequent training performance has been very stable and consistent across studies in one with 25 sessions [15] or in another with 105 sessions [18], with the errors being significantly below chance. The performances at short-term memory tests are also similar after 12–14 training sessions [15] or after 96–100 training sessions [18]. In middle-aged animals, subsequent training performance is also comparable in a group with prior training [15] and in a group without prior training [26]. Prior training does not improve long-term memory or intermediate-term memory in middle-aged animals [15]. Together, these would suggest that the prior training effect on short-term memory is more pronounced in aged rats, compared to young rats, and the effect on intermediate-term memory is more pronounced in aged rats, compared to middle-aged rats.

With these profound effects in aged animals, it is likely that prior training affects multiple pathways involved in memory function. For example, sensory and cognitive stimulation are associated with changes in a myriad of molecular and cellular mediators, such as expression of brain-derived neurotrophic factors, nerve growth factors and neurogenesis (review in [81]). While most of these changes are seen in young animals, there is evidence for gene expression changes in old animals. Prior lifelong wheel running improves long-term memory in a brief water maze test at old age and increases signal intensities in genes associated with synaptic transmission and energy metabolism [82]. Activities in prior training in this or our earlier studies [15, 26, 83] are relatively mild, compared to intensive exercise that is shown to improve learning or memory (e.g., wheel running for 1.5 km per day in [82] or treadmill running for approximately 330 m per day for 100 days in [84]). Environmental enrichment has also been shown to improve cognition in aging, although it typically involves more objects for prolonged interaction [85–87] than two landmarks and sandwells in this study that animals tend to habituate to. It is conceivable that cognitive stimulation plays a crucial role in the prior training effect seen here. The stimulation includes learning of the procedural aspects of the task as discussed above, formation and consolidation of the spatial map that enables assimilation in the case of schema learning [88, 89], rapid updating and maintaining of daily information on reward locations, and occasional exposure to novelty that modulated memory persistence [15, 18, 26, 30, 83]. Studies have shown that ‘training’, as opposed to activity-only control, is critical for improvement in navigation or memory abilities in aging [90, 91].

At the brain circuit level, it is likely that such prior training benefits would recruit the frontal or cortical network. The time course of the prior training would engage systems consolidation [16] that is shown to involve prefrontal cortex [92]. Prior training also enables the formation of the spatial knowledge and learning rules that is shown to require neural transmissions in the prefrontal cortex [88] and anterior cingulate cortex [89, 93]. A recent study suggests that proteins in the prefrontal cortex can explain cognitive resilience in aging. Analyses of human clinical testing and prototypic peptides show that higher levels of synaptic plasticity-related proteins, such SNAP25, SYT12, and VGF, and mitochondrial proteins, such as NDUFA, are associated with slower cognitive decline [94].

### Preserved learning, motor, and motivation in aging

The task acquisition ability is unchanged in older animals in this ADMP task, which is evident by errors made during training being below chance. No learning deficit was observed in our previous study evaluating age-related decline in this ADMP task in middle-aged rats [15, 26]. To our surprise, errors that older rats made at retrieval are already below chance from early training sessions in the no prior training group. This could be due to preference in using a matching rule in finding more rewards in the same location after a delay, consistent with intact acquisition of delayed matching-to-place task in the water maze in aged rats [95]. Our task requires the animals to visit the matching location between encoding and retrieval to obtain more rewards. As there are more than 1 non-rewarded location at the retrieval trials or probe tests, this paradigm is not currently designed for non-matching tests that are seen in 2-choice tasks [96, 97]. Through increasing difficulty in a radial maze task, a delayed-non-matching-to-sample task that involves both spatial and working memory can be a sensitive assay to detect age-dependent memory decline [98].

Efficiency in retrieval during rewarded learning is also intact in older rats. For the group without prior training, the steady linear decrease of latencies in retrieval is comparable with what has been observed in young and middle-aged rats [15, 26], indicating that aging did not affect the motor and learning capacity of the rats in this ADMP task. It is consistent with previous studies showing that sensorimotor abilities, exploration of a complex environment, or spontaneous locomotor activities during the light phase are intact in old rats [44, 47, 99]. Intact latencies also imply that motivation and motor in performing this task is intact in old rats as previously observed [47]. These findings are different from an age-dependent impairment in latencies in a reference task in the water maze and slower swimming speed in ex-breeder female rats [37]. It is likely that increase in physical demanding of the task, further advancement in aging, sex, and history of the animals contribute to decline in motor or in efficiency in task performance in aging.

Frequent handling during training in this study would likely lead to habituation to handling. Animals also received weekly handling and weighing from the age of 3 months onwards, while the prior trained group received additional handing on the training days in younger age. Frequent transportation on a daily basis during training would likely lead to habituation to transportation. Our attempt to address the necessity of training in contributing to cognitive benefits can been seen in a recent study using a mouse model of Alzheimer’s disease with the wildtype littermates [100]. Prior training, compared to handling control, improves performance efficiency and accuracy in midlife [100]. The ADMP training in the current study was done in the light phase. Sleep disturbance might occur. While the sleep quality and patterns were not monitored in this study, rats were often asleep after performing the short daily task. As sleep disturbance has negative impacts on memory [101, 102], it is likely that the benefit from prior training observed in this study is underestimated if sleep disturbance had occurred at the younger age.

## CONCLUSION

Overall, our findings suggest a selective impairment in encoding for long-term memory formation in early aging and an additional impairment in consolidation in later aging. Learning ability, short-term memories, motor and motivation functions remain intact in older age, suggesting a phase when memory-associated processes are compromised before apparent navigation or learning deficits in advanced aging [103]. Prior training shows profound benefits in cognitive aging and it can provide a translatable model to simulate human cognition which is built upon lifelong experiences.

## MATERIALS AND METHODS

### Animals

Adult male Lister Hooded rats (Charles River, 200–225 g on arrival, *n* = 26) were group-housed at 3–4 rats per cage throughout their lives. The cages (L46 × W37 × H20 cm) were lined with bedding (a mixture of sawdust (Datesand) and wood shavings (DBM Ltd UK), at approximately 1:1 ratio and placed in a colony room with regulated temperature (20–22°C) and humidity (40–60%). The room was under a 12 h light/dark cycle (light onset 7.00AM) and behavioral training and testing was conducted during the light phase (between 9.00AM and 5.00 PM). Food (Diet #801700, DBM Ltd UK) and water were available *ad libitum* except that during training and test sessions food was restricted to 80 g to 100 g per cage of 4 rats per day to maintain their body weight at around 90–95% of free-feeding weight. The food amount was adjusted based on the animals’ body weight and age. All rats were obtained from the same supplier at highly similar weight/age. They were aged in the same colony room in our research facility. They were handled at least once per week during the 20-month period by the experimenters to reduce possible stress and to keep the animals in contact with the experimenters. For training and testing, cages were placed on a trolley and transported to the experimental room at about 1 hour before the beginning of the experiments and returned to the colony room at about 1 hour after the behavioral procedures. They would remain in the colony room overnight and during non-experimental time. Cages were changed once per week. If the cage change fell on days with behavioral procedures, it would be done at > 1 h after the behavioral session was concluded to reduce disturbance.

### Experimental design

This study involved 2 groups of aged rats (19 to 23-months-old), with or without prior training at younger age. The first group, without prior early-life training, received training (Figure 1A) when they were at the age of 19 months. The interleaved training and testing continued till they were 23-month-old (*n* = 13, Figure 1B–1E). The second group, with prior training, received training and testing at the age of 3 to 5 months, 11 to 13 months, and finally 19 to 23 months (*n* = 13, Figure 1F–1I). The performance of group 2 at 2 younger age points were previously reported in Gros and Wang, 2018 (young: 12 training sessions, 7 probe tests, 6 interleaving sessions; mid-age: 12 training sessions, 13 probe tests, 14 interleaving sessions). Both groups of rats at 19 months-old received 12 initial training sessions and 10 interleaving training sessions that were among 11 (for group 1 and 12 for group 2) encoding-probe sessions to evaluate their memory persistence.

### Apparatus for the appetitive delayed matching-to-place memory (ADMP) task

Behavioral experiments were conducted in an event arena (135 × 135 × 40 cm, made of clear Plexiglas walls and white Plexiglas floor, Figure 1A) lined with around 2 cm sawdust and contained 2 intra-maze landmarks, as previously described [15]. Four start boxes (30 × 25 × 30 cm) were placed in the center of each wall. They were covered with red films that darkened the box and equipped with automated doors under the control of the experimenter. Chocolate-flavored food pellets (Supreme Mini Treats™, ref: F05472, Bio-Serv) were used as food rewards (0.5 g per reward). Plexiglas sandwells (6 cm diameter, 4 cm depth) could be inserted into the floor of the arena at different locations. Sandwells were filled with bird sand mixing with 5% of ground pellets. Four food pellets were embedded at the bottom of every sandwell and kept out of reach of the animals by a metal mesh divider. These were designed to keep olfactory cues more consistent among all sandwells. The arena was placed in a rectangular laboratory room with extra-maze visual cues on 3 walls and 1 curtain.

### Box and substrates for inducing novelty

A square Plexiglas box (100 × 100 × 40 cm) with opaque white walls was used. Small substrates were placed on the floor to encourage exploration. To introduce novelty, different materials with distinct shapes, sizes, and textures, such as small aquarium pebbles, polished stones, plastic sealing clips, and small bricks, were placed on the floor of the square box (an example in Figure 3D). Rats have been shown to rapidly sense the textures in the environment [104] or respond to texture novelty [105].

### Behavioral procedures

The ADMP task was designed to gauge the animals’ spatial memory [18, 106] and also to simulate our daily experience of learning a location of interest and navigate to the same location after a delay (e.g., park a bike and come back to retrieval it afterwards). Rats were trained to locate the reward location in an open arena and then to use the encoded information to find and obtain more rewards after a delay when they would face multiple choices. This task has been used and replicated in previous studies [15, 18, 25, 26, 30, 31, 106]. In brief, rats were first habituated to the experimenters, to the event arena apparatus and procedures, before being trained to perform the ADMP task in the event arena. Theses pre-training procedures are designed to reduce stress and to familiarize animals with the environment. There were 12 initial training sessions to train the animals with the matching rule of the task. We then examined memory performance at various delayed durations in probe tests with different encoding strength and with or without novelty after encoding. These were to assess their short-term, intermediate-term and long-term memory persistence and to determine if peri-encoding novelty could facilitate memory persistence.

#### Habituation

Phase 1: habituation to the experimenter. Rats were handled every day for 5 days to habituate them to the experimenter to reduce stress. Daily body weight was measured to establish the baseline of weight gain under normal feeding. Phase 2: habituation to the sandwell and food pellets. Rats received limited food daily from now on to maintain the body weight at 90–95% of free-feeding weight. Sandwells with chocolate-flavored pellets were placed in their home cage for 30 min daily for 2 days. They naturally explored and dug through the sand and found and ate the pellets. Phase 3: habituation to the sandwell in the event arena. Rats were then habituated to digging the sandwells in the event arena. First, rats explored a quarter of the event arena (divided by removable inserts) with a sandwell containing 4 pellets (one pellet on top and three pellets in the middle of the sandwell). They would receive 4 trials and hence explored 4 quadrants of the arena. Second, rats explored half of the event arena with a sandwell containing 4 pellets (one pellet on top and three pellets in the middle of the sandwell). They would receive 2 trials and hence explored 2 halves of the arena. Finally, the rats explored the whole event arena with a sandwell placed at the center of the arena containing 4 pellets (one pellet on top and three pellets in the middle of the sandwell). They would explore the arena voluntarily, find the pellet and carry it to the start box to eat. Each habituation trial stopped when they found and ate all the 4 pellets or capped at 15 minutes.

#### Training

Rats were trained in the event arena for 12 sessions at 5, 6 days per week. A daily training session consisted of a sampling trial for memory encoding followed by a choice trial for memory retrieval about 40 min later (Figure 1A). During the encoding trial, one rewarded sandwell was placed in the arena at a particular location and constituted the opportunity for each rat to encode where the food was available on that day. The rewarded location (e.g., far from or near the start box) on a given day was counter-balanced across all rats to avoid bias toward certain regions of the arena. The rewarded location for a given rat was also counter-balanced across training sessions to avoid preference of certain regions of the arena. The rewarded sandwell location and the start box (North, East, South, West) were changed across days to encourage the animal to encode a new location on different days, simulating our daily experience of locating and retrieving where we park a vehicle or place an object. Rats were given a pellet in the start box at the beginning of each trial to be accustomed with eating at the start box. After about 30 sec when the pellet was consumed, the experimenter would remotely open the door without being seen by the animal for the rest of the trial. After the door opened, rats explored the arena, found the sandwell location, dug to find the hidden reward, and then returned to the start box to eat the pellet. The rats repeated these procedures until they collected 3 pellets. The door would be closed, and the trial ended.

At the retrieval trial, 5 different sandwell locations were present but only the sandwell location that matched the rewarded place at the encoding trial would contain rewards. This was designed to train the animal to use a matching rule to find the reward location. If the rewarded location during the encoding trial was remembered, the animal would make minimal errors in digging at non-matching location before return to the matching location to find more rewards. The trial ended after the rats had retrieved and eaten 3 pellets.

Rats were trained sequentially in batches of 6–8 rats per batch. In batch 1, rat 1 would receive an encoding trial and then return to the home cage. Next, rat 2 would receive an encoding trial with the rewarded sandwell in a different location from rat 1 and then return to the home cage, so on and so forth. The encoding phase typically took about 40 minutes (5 min per rats). Rat 1 would then receive the retrieval trial and then return to the home cage. Same for rat 2 and so on and so forth. The smell-based navigation strategy was reduced or prevented by (1) using different rewarded locations between trials (i.e., the same rewarded location was not used in 2 consecutive trials), (2) training different rats in between encoding and retrieval (or probe), (3) mixing arena bedding between trials, (4) mixing grounded reward in the sandwell, and (5) adding rewards at the bottom of the sandwell with in an inaccessible compartment (also see description in [18]). All animals spontaneously engaged with the training or retrieval trials, so no intervention was required to enable the animals to respond. The prior trained group was conducted before the other group. We ensured that the quality and consistency of the cross-group comparison was reliable by using the same arena, colony room, animal supplier, methods of housing, methods of transportation, and the same behavioral protocols. The comparability between groups was confirmed toward the last block of training. Both groups at block 4 of training showed similarity in errors (Bonferroni’s multiple comparisons test *p* > 0.99) and in latencies (*p* > 0.99).

#### Probe tests

After 12 initial training sessions, rats received various encoding-probe test sessions to assess their memory persistence. A test session was consisted of an encoding trial with a single rewarded sandwell, followed by a 60-sec probe trial with 5 non-rewarded sandwells at certain delays. To assess their short-term memory, the delay was 1 h (Figure 2). To assess their intermediate-term memory, the delay was 6 h (Figure 3). To assess their long-term memory, the delay was 24 h (Figure 4). One of these 5 non-rewarded sandwells would be at the matching location to the rewarded place at the encoding trial. The animals would explore the arena and dig sandwells. After 60 sec, the experimenters placed 1 pellet at top of the matching sandwell and 2 pellets at the bottom of it, so rats could find, retrieve, and eat the pellets. This was to avoid weakening of using the matching rule to search after non-rewarded probe tests. A reactivation trial involved an animal exploring the arena with a non-rewarded sandwell at the matching location to the rewarded location at encoding (Figure 5). The trial was ended when the rat returned to the start box after exploring the arena and digging the sandwell. Counterbalancing between paired conditions (e.g., with and without memory-modulating events after encoding) was performed.

#### Memory-modulating events

To evaluate if novelty (an example, see Figure 3D) after encoding would facilitate memory persistence, rats would receive an encoding trial, and approx. 30 min later a 5-min trial of spontaneous exploration in a novel box. This would be run in a counterbalanced order with no modulating event (i.e., half of the rats received encoding + no novelty + probe before they were retrained and received encoding + novelty + probe, while the other half received the reversed order). To evaluate if exploration of an encoding zone would facilitate long-term memory, rats would receive an encoding trial, and approx. 30 min later a 5-min trial during which a rat could freely explore the zone of the arena (approx. 45 × 55 cm, enclosed with clear plexiglass walls at 40 cm height) that centered around of the previously rewarded sandwell location. For this, plexiglass walls were installed into the arena to limit the area containing the food location. To evaluate if strengthening the rewarded encoding would facilitate long-term memory, rats would receive an encoding trial, and approx. 30 min later another encoding trial.

#### Interleaving training sessions and test sequence

These were identical to the initial training trials except that 1 such session was introduced between 2 probe tests. One exception was 2 consecutive sessions at session 18– 19 and 1 omission at session 28 in the prior group. The goal of interleaving training was to prevent reduction in sandwell digging time and retain the use of the matching rule after non-rewarded probe. The sequence of tests was: long-term memory after strong encoding, after strong encoding followed by a novel box or encoding zone (counterbalanced), after 2 strong encodings; intermediate-term memory after strong encoding, after weak encoding (with or without novelty, counterbalanced); short-term memory after weak encoding; reactivation (with or without box, counterbalanced) and no reactivation control. For the prior training group, the last session was a weak encoding trial followed by a short-term memory test with mismatched start box locations. The consistency of memory performance in this task has been examined in previous studies regardless of the test sequence [15, 18, 30, 31].

### Behavioral measurement and analysis

During training, how many non-matching (i.e., wrong) sandwells visited by rats before they dug in the correct sandwell during the retrieval trial would constitute errors. This index would reflect the accuracy in memory retrieval. Latencies (in sec) to find the pellets in the rewarded sandwell in the retrieval trial were also measured to reflect the efficiency of retrieval.

To assess memory at probe tests, the time that rats spent digging (moving sand from the sandwell with forepaws) in the 5 sandwells was recorded for the first 60 sec of the trial. Sniffing or touching the sandwell with the nose was not included in the digging time. A custom-built LabView timer was used to record the digging durations and latencies. The correct digging percentage was calculated by the percentage of time digging at the correct (i.e., matching to encoding) location over the total digging time. The wrong digging percentage was calculated by the percentage of averaged digging time at the 4 non-matching locations over the total digging time. The experimenters were blind to the encoding conditions when measuring and recording the digging duration.

### Statistical analysis

#### Training analysis

Data were presented in 4 blocks of 3 training sessions per block. Data were averaged across 3 sessions per animal per block for statistical analysis and then averaged across animals for figures. The number of errors was analyzed using repeated-measures one-way ANOVA across blocks followed by two-tailed, one sample *t*-tests to compare each block with the chance level. The chance level of errors was 2, and a score of 0 would mean that the animal dug at the matching location without digging at other locations. The latency to obtain all rewards was analyzed using repeated-measures one-way ANOVA across blocks. The training effect in latency reduction has been shown to be robust with a statistical power of 1 and a large effect size of Cohen’s d > 1.54 [15, 30]. Comparisons between groups were analyzed using repeated-measures two-way ANOVA followed by Bonferroni’s multiple comparisons tests. Comparisons between ages in priorly trained animals were analyzed using repeated-measures two-way ANOVA followed by Tukey tests.

#### Test analysis

Data were averaged across animals within each encoding-probe condition and were presented as mean ± SD in figures. The percentage of digging in the correct location was compared with the mean percentage of digging in wrong locations using two-tailed paired *t*-tests. To evaluate if performance was different between paired conditions (e.g., without vs. with novelty, encoding zone exploration, or second encoding trial after encoding), the percentages of correct digging was compared using a two-tailed paired *t*-test. The chance level for the percentage of correct digging was 20 %. To evaluate the benefit of prior training, between-group comparisons were done by two-tailed, independent two-sample *t*-tests. Comparisons between groups in Figure 3 and Figure 5 were analyzed using repeated-measures two-way ANOVA. Short-term memory persistence in this task has been shown to be robust with a statistical power of 1 and a large effect size of Cohen’s d > 0.94 [15, 30]. Parametric tests were used as the data did not violate a normal distribution (Shapiro-Wilk test). A power calculation based on our previous papers in young and mid-aged rats [15, 18, 30] would suggest *n* ≥ 8 rats per group (based on latency) to achieve good learning and *n* ≥ 12 per group (based on long-term memory) to achieve good memory. For all statistical tests of the training data and most of the test data, the size of the population was *n* = 13 for both groups. For the reactivation probe tests done around 23-month-old, the size of the population for group 1 (no prior training) was reduced to *n* = 10 as 3 rats passed away. Statistical significance was set at *p* < 0.05. All statistical analysis was done using SPSS Statistics 22 (IBM).
